# Effects of Task Constraint Manipulation on Physical, Physiological and Technical–Tactical Demands of Small-Sided Games in Soccer: A Systematic Review

**DOI:** 10.3390/jfmk11030354

**Published:** 2026-09-05

**Authors:** Pakorn Chootsungnoen, Wirat Sonchan, Phornpot Chainok, Pornchai Leenoi

**Affiliations:** 1 Faculty of Sports Science, Kasem Bundit University, 77 Romklao Road, Minburi, Bangkok 10510, Thailand; pakorn.cho@kbu.ac.th (P.C.); pornchai.lee@kbu.ac.th (P.L.); 2 Faculty of Sport Science, Burapha University, Chonburi 20131, Thailand; wirats@go.buu.ac.th

**Keywords:** small-sided games, task constraints, football, training load, transition games, floater

## Abstract

**Background:** Small-sided games (SSGs) are widely used in soccer training to develop physical, physiological, and technical–tactical capacities under game-representative conditions. Although task constraints have been investigated individually, evidence integrating their effects across major categories remains limited. This systematic review synthesized the effects of six task-constraint domains on SSG demands in association football. **Methods:** Following PRISMA 2020 guidelines, systematic searches were conducted in PubMed, SPORTDiscus, Web of Science, and Scopus. Forty studies published between 2015 and 2025 met the eligibility criteria and were categorized into six task-constraint domains: pitch size or area per player (*n* = 13), player number (*n* = 8), goalkeeper inclusion (*n* = 6), goal type (*n* = 5), numerical imbalance and floater players (*n* = 8), and transition games (*n* = 6). The review protocol was prospectively registered in PROSPERO. **Results:** Larger pitch areas consistently increased high-speed running but were insufficient to reproduce match-level sprint demands. Goalkeeper exclusion increased physiological intensity, particularly in smaller formats, whereas mini-goals reduced external locomotor load while maintaining comparable internal responses. Numerical disadvantage increased sprinting and high-intensity running demands for outnumbered players, while floaters experienced substantially lower physical loads than regular players. Transition games elicited the greatest locomotor demands, exceeding match values for high-speed running and sprinting across several playing positions. **Conclusions:** No single SSG format adequately addresses all match-relevant physical demands. Accordingly, practitioners should systematically manipulate and combine task constraints in accordance with specific training objectives. These findings provide evidence-informed guidance for optimizing SSG design to elicit targeted physical, physiological, and technical–tactical adaptations.

## 1. Introduction

Small-sided games (SSGs) have become a cornerstone of contemporary soccer training methodology, providing an ecologically valid environment in which physical conditioning and technical–tactical development are pursued concurrently [1,2]. Reducing pitch dimensions and player numbers relative to the 11v11 format increases the frequency of individual ball involvements and technical actions while altering locomotor and physiological demands according to the imposed task constraints. Consequently, SSGs provide a representative, sport-specific training stimulus that enables the concurrent development of physical, physiological, and technical–tactical capacities within an integrated training task [1,2]. A fundamental principle underpinning SSG design is that task constraints—the rules, spatial dimensions, and numerical configurations that coaches systematically manipulate—channel players towards desired physical and tactical behaviors [2]. According to the ecological dynamic’s framework, constraints interact to shape the affordances available to players and consequently, the emergent movement and decision-making patterns during game play.

Evidence integration is further constrained by variation in outcome definitions, participant characteristics, and SSG design across studies [3,4,5]. Furthermore, different constraints selectively modify locomotor, physiological, and tactical demands [6,7]. Accordingly, transition games are not considered a single task constraint in the present review but rather a composite SSG format in which multiple task constraints are manipulated simultaneously. They were nevertheless retained as a separate analytical category because the existing literature commonly investigates transition games as a distinct training format with shared design characteristics, allowing their evidence to be synthesized separately from studies examining isolated task-constraint manipulations [8,9].

Six primary categories of SSG constraints have received scientific attention: (1) pitch size and area per player, (2) number of players, (3) goalkeeper presence and role, (4) goal type, (5) numerical imbalance and floater players and (6) transition-game formats. Prior systematic reviews have addressed single constraint categories, most commonly pitch size [3] without integrating the broader constraint landscape. As a result, existing research remains disorganized, thereby limiting a thorough understanding of how varied constraint modifications affect the multidimensional demands of SSGs [3]. This limitation is increasingly important given the widespread use of global positioning systems (GPSs), local positioning systems (LPSs) and inertial measurement units (IMUs), which have substantially expanded the quantity and precision of workload and performance data available for analysis.

A comprehensive synthesis of the literature is necessary to synthesize evidence across key categories of task constraints, identify consistent patterns in players’ physical, physiological and technical–tactical responses and provide practitioners with evidence-based recommendations for designing SSGs that correspond with specific training objectives and player development requirements. The purpose of this systematic review was therefore to comprehensively synthesize evidence on the effects of task-constraint manipulation across all six identified categories on physical, physiological and technical–tactical demands of SSGs in association football. A secondary aim was to provide evidence-based recommendations for practitioners.

## 2. Materials and Methods

### 2.1. Search Strategy and Eligibility

This review was conducted in accordance with the Preferred Reporting Items for Systematic Reviews and Meta-Analyses (PRISMA) 2020 Statement [10]. Electronic searches were performed in PubMed/MEDLINE, SPORTDiscus, Web of Science Core Collection and Scopus from inception to December 2024. The search strategy combined controlled vocabulary (where applicable) and free-text terms related to SSGs (“small-sided game*”, “transition game*”), football/soccer, task constraints (“pitch size”, “goalkeeper”, “mini-goal*”, “floater*”, “numerical imbalance”, “player number*”) and outcome measures (“physical demand*”, “training load”, “GPS”, “heart rate”, “tactical*”). Records retrieved from all four databases were consolidated into a single dataset and cross-checked for duplicates using bibliographic information, including title, authors, publication year, and DOI. A total of 98 duplicate records were identified and removed before title and abstract screening.

Following the initial database search and screening process, backward citation tracking of the reference lists of all eligible studies and relevant systematic reviews was performed to identify additional potentially eligible studies. The final citation-tracking search was completed on 2 August 2026. Studies published in 2025 that met the predefined eligibility criteria were identified through this prespecified citation-tracking procedure rather than the original database searches and were subsequently screened and included in the review. The review protocol was prospectively registered in PROSPERO (CRD420261433575) before data extraction, with the final citation-tracking search completed on 2 August 2026. The complete database-specific search strategies for PubMed/MEDLINE, SPORTDiscus, Web of Science Core Collection and Scopus, including Boolean operators, truncation symbols, field restrictions and controlled vocabulary (where applicable), are provided in Supplementary File S1.

Studies were included if they: (1) involved male football (soccer) players of any age or competitive level; (2) employed an SSG or transition-game format; (3) manipulated at least one task constraint; (4) reported physical, physiological, or technical–tactical outcomes; (5) used an experimental or observational design with ≥10 participants; and (6) were published in a peer-reviewed journal. Studies involving sports other than football (e.g., futsal), duplicate datasets, conference abstracts, review articles, or meta-analyses were excluded. These eligibility criteria were defined a priori in accordance with the review objective and the established SSG literature [1,2] to capture empirical studies examining how task-constraint manipulation influences physical, physiological, or technical–tactical responses in association football. Studies involving fewer than 10 participants were excluded to minimize the inclusion of very small case-series studies. The ≥10-participant threshold was established a priori as a pragmatic criterion to ensure a minimum level of methodological robustness while maintaining broad coverage of the available literature. Experimental, longitudinal and observational study designs were eligible to capture both controlled and applied SSG settings.

Two reviewers independently screened the titles and abstracts of all retrieved records according to the predefined eligibility criteria. Full-text articles considered potentially eligible were subsequently assessed independently by the same reviewers. Any disagreements regarding study eligibility were resolved through discussion and consensus. Inter-rater agreement statistics were not calculated because disagreements were resolved through consensus throughout the review process.

### 2.2. Data Extraction and Quality Assessment

Data extraction was performed independently by two reviewers using a standardized data extraction form developed a priori. The extracted information included the first author, publication year, participant characteristics, SSG format, manipulated task constraint(s), outcome measures and principal findings. The extracted data were cross-checked for accuracy and completeness and any discrepancies were resolved through discussion and consensus before the methodological quality assessment was undertaken. Methodological quality was assessed using a modified Downs and Black checklist [11] adapted for SSG research. The checklist evaluated four methodological domains: reporting quality (7 points), external validity (3 points), internal validity related to bias (7 points) and internal validity related to confounding (6 points), yielding a maximum score of 27 points. Studies were classified as good (≥17 points), fair (11–16 points), or poor (≤10 points).

The modified Downs and Black checklist was selected because it provides a comprehensive evaluation of methodological quality across key domains, including reporting quality, external validity, internal validity (bias) and confounding, and has been widely applied in previous systematic reviews of soccer small-sided games. Given the methodological heterogeneity of the included studies, encompassing experimental, longitudinal and observational designs, a single appraisal framework was considered the most appropriate approach to ensure a consistent and comparable assessment of methodological quality across all eligible studies.

The checklist was adapted by excluding items that were not applicable to the predominantly non-randomized and acute study designs included in this review while retaining those relevant to the assessment of methodological rigor. These modifications enhanced the applicability of the instrument without compromising its conceptual framework. A detailed description of the modifications made to the original Downs and Black checklist, together with the rationale for each modification, is provided in Supplementary Table S1. Although design-specific appraisal tools, such as the AXIS and JBI Critical Appraisal Checklists, may offer greater specificity for observational studies, the modified Downs and Black checklist was considered the most appropriate instrument for this review because it enabled a standardized and directly comparable evaluation across the diverse study designs included.

### 2.3. Data Synthesis

A meta-analysis was not undertaken because the included studies were not sufficiently comparable to support meaningful quantitative synthesis. Substantial heterogeneity was observed in study design (experimental, longitudinal and observational), task-constraint manipulations (e.g., pitch dimensions, player number, goalkeeper conditions, goal type, numerical imbalance and transition-game formats), participant characteristics (age, competitive level and playing standard) and SSG configurations (e.g., game format, duration, work-to-rest ratio and area per player). Additional variability existed in the outcomes assessed, measurement methods (e.g., GPS-derived metrics, heart-rate responses, ratings of perceived exertion and technical–tactical variables) and the operational definitions of key variables, particularly high-speed running and sprinting thresholds. Furthermore, the studies reported outcomes using different statistical approaches and effect estimates, limiting direct comparability across investigations. Consequently, a narrative synthesis was conducted to summarize the evidence according to the primary task-constraint category while considering methodological quality, consistency of findings and the strength of the available evidence.

## 3. Results

### 3.1. Search Results and Study Quality

**Figure 1:**
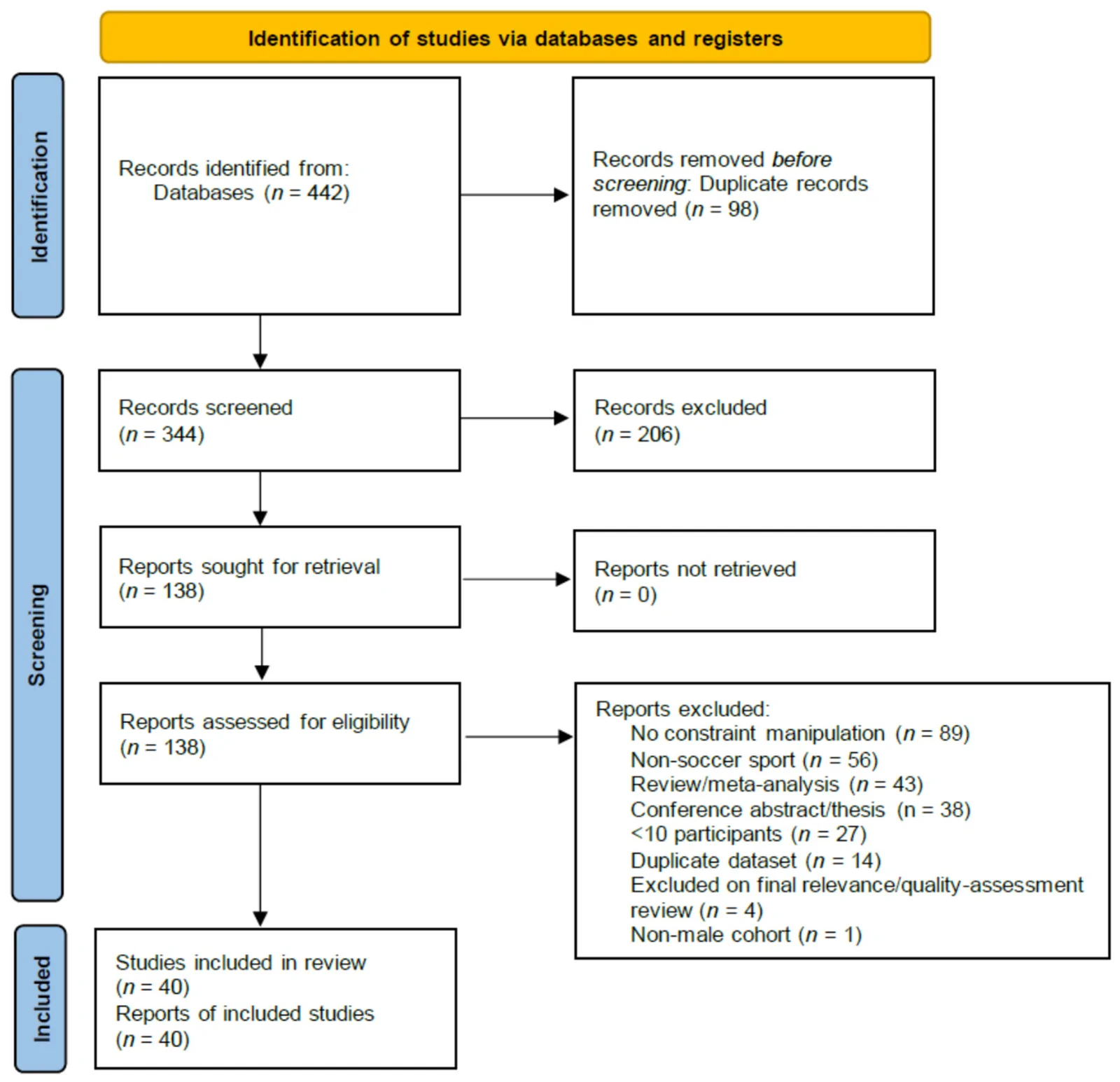
PRISMA flow diagram of the study selection process (template by Page et al. [10]).

The literature search identified 442 records from the four databases, of which 98 were duplicates and were removed prior to screening (Figure 1). Following title/abstract screening of the remaining 344 records, 206 were excluded and 138 reports were sought for retrieval (all 138 were successfully retrieved). Of the 138 reports assessed for eligibility, 40 met the predefined inclusion criteria and were included in the review. Based on the modified Downs and Black checklist, 29 studies were classified as good quality (≥17/27), whereas the remaining eleven studies were rated as fair quality (11–16/27) and no studies were rated poor (Table 1). The most frequently identified methodological limitations were limited external validity, primarily due to the predominance of male participants and single-institution study samples, together with insufficient control of potential confounding factors, including participants’ training history and fitness level.

### 3.2. Pitch Size and Area per Player

Thirteen studies examined pitch dimensions or area per player (ApP) on the physical, physiological and technical–tactical demands of SSGs (Table 2). Overall, increasing ApP consistently elevated external load, resulting in greater total distance covered, high-speed running (HSR; typically >14 km·h^−1^) and in most studies, higher mean heart rate and ratings of perceived exertion (RPE) [3]. Castillo-Rodríguez et al. [36] reported that an ApP of approximately 150–250 m^2^ per player was required to reproduce the physical and physiological demands observed during under-19 match play, whereas smaller playing areas failed to elicit sufficient high-speed running and sprinting stimuli.

Castillo-Rodríguez et al. [36] identified 150–250 m^2^/player as the threshold for replicating U19 match physical and physiological demands; below this, high-speed and sprint requirements were inadequately stimulated. Similarly, de Dios-Álvarez et al. [37] demonstrated that the most demanding one-minute worst-case scenario (WCS) recorded during official matches consistently exceeded those observed across all SSG formats, irrespective of ApP. Notably, SSGs with an ApP below 100 m^2^ per player increased mechanical loading through more frequent accelerations and decelerations while substantially underestimating sprint-related demands.

Li et al. [33] further demonstrated that when the area per player (ApP) was maintained at 80 m^2^ across 3v3, 5v5 and 8v8 formats, internal load variables did not differ significantly despite variations in external load, supporting the premise that ApP is a primary determinant of physiological intensity. Riboli et al. [6] also identified a significant interaction between ApP and goalkeeper presence, reporting that substantially larger playing areas were required to replicate match demands when goalkeepers were included (187 m^2^ per player for total distance) than when they were excluded (115 m^2^ per player). Santos et al. [30] identified age-related differences, noting that the impact of pitch size on metabolic power was evident just in U-12 players, indicating that younger athletes may have a diminished ability to use greater physical capabilities. The findings described above are derived predominantly from studies examining acute physical and physiological responses during individual SSG sessions. In contrast, evidence regarding chronic training adaptations remains limited. Compared to the majority of acute studies, Wang et al. [9] provided longitudinal evidence from an 8-week randomized controlled trial, demonstrating that training on expanded pitches (length-to-width ratio = 2.0) resulted in greater improvements in sprint performance compared to training on square pitch configurations.

Overall, the evidence for the effects of pitch size and area per player is supported predominantly by studies classified as good methodological quality, with the remaining fair-quality studies reporting generally consistent findings. This consistency across studies with different methodological quality strengthens confidence in the conclusion that increasing the area per player increases external locomotor demands, although larger playing areas remain insufficient to fully reproduce match-level sprint demands.

### 3.3. Number of Players

Eight studies manipulated player numbers as the primary constraint (Table 3). Overall, smaller game formats were associated with greater mechanical and physiological demands, whereas larger formats elicited higher high-speed running. Rebelo et al. [15] demonstrated a clear dose–response, in which 4v4 formats produced significantly more accelerations and decelerations and higher blood lactate concentrations than 8v8, while 8v8 elicited greater very-high-speed running. Similarly, Lacome et al. [4] analyzed 249 SSG sessions across 4v4 to 10v10 formats in elite players and reported that no format fully replicated the high-speed running demands of official match play. Smaller formats produced greater mechanical loads, whereas larger formats generated higher high-speed running, with significant position-specific differences observed across playing positions.

In youth contexts, Castellano et al. [16,17] consistently showed that relative pitch area exerted stronger influence than player number on locomotor demands in large-sided game formats, although increasing the number of players was associated with higher maximum running speeds. Farhani et al. [44] reported that continuous 12 min SSG bouts resulted in better technical performance and lower mood disturbance than fragmented training bouts. Most evidence regarding the influence of player number was derived from studies classified as good methodological quality, whereas the limited fair-quality evidence reported comparable findings, suggesting a consistent effect across studies with different methodological quality.

### 3.4. Effects of Goalkeeper-Related Constraints

Six studies examined the effects of goalkeeper (GK) presence, absence, or role modification on the physical, physiological and technical–tactical demands of SSGs (Table 4) Köklü et al. [45] demonstrated across 2v2, 3v3 and 4v4 formats that removing the goalkeeper consistently elicited higher heart rate, blood lactate concentration, ratings of perceived exertion (RPE) and running distances across all movement intensity zones. Hulka et al. [19] reported that this effect was moderated by pitch size, with goalkeeper presence reducing the workload of outfield players only in smaller pitch configurations. Santos et al. [29] further showed that goalkeeper format (possession versus goalkeeper-scoring) exerted a greater influence on physical load than pitch size. In a longitudinal study, Jumareng et al. [41] reported that goalkeeper verbal encouragement during 3v3 SSGs produced greater improvements in physical and technical performance following an 8-week training intervention. The available evidence was predominantly supported by studies of good methodological quality, although the relatively small number of eligible studies warrants cautious interpretation.

### 3.5. Effects of Goal-Type Constraints

Five studies examined the effects of goal type, including regular goals, mini-goals and possession-play formats, on the physical, physiological and technical–tactical demands of SSGs (Table 5). Overall, goal type influenced external load, physiological responses and tactical behavior, with the magnitude of these effects depending on game format. Bujalance-Moreno et al. [28] showed mini-goals produced significantly lower external load (total distance, HSR, maximal speed, accelerations ≥ 2.5 m/s^2^) while eliciting similar internal load and neuromuscular fatigue compared with possession-play formats. González-Rodenas et al. [14] demonstrated an interaction between goal type and player number with small goals eliciting higher heart rate responses than regular goals during 4v4 SSGs but not during 6v6 formats. Castellano et al. [18] further reported that small-goal formats resulted in greater defensive team dispersion than regular goal formats with goalkeepers. Evidence regarding goal type was derived from both good- and fair-quality studies. Despite this methodological variation, the findings were generally consistent across studies.

### 3.6. Effects of Numerical Imbalance and Floater Use

Eight studies examined the effects of numerical imbalance and the use of floater players on the physical, physiological and technical–tactical demands of SSGs (Table 6). Guard et al. [34] reported that numerically overloaded teams (4v6) demonstrated significantly higher mean heart rate (84.4% vs. 80.4% HRmax) than underloaded teams. Nunes et al. [27] found that greater numerical inferiority (4v6) was associated with increased sprinting activity and tactical action frequency, whereas greater numerical superiority (4v2) resulted in more time spent walking. Tactically, Padilha et al. [21] further reported that the inclusion of floater players increased offensive width and improved defensive compactness. Rábano-Muñoz et al. [8] and Lozano et al. [26] consistently found that floater players performed substantially lower physical workloads than regular players. Based on these findings, Lozano et al. [26] proposed a progressive training framework in which floater and regular-player roles were used to gradually modify external locomotor demands. However, this framework should be interpreted as a proposed load-progression approach rather than as evidence supporting the effectiveness or safety of a return-to-play strategy. Most studies investigating numerical imbalance and floater players were rated as good methodological quality, providing moderate confidence in the consistency of the reported findings.

### 3.7. Transition Games as a Composite Small-Sided Game Format

Six studies examined the effects of transition games (TGs) on the physical and physiological demands of soccer players (Table 7). Overall, transition-game formats frequently elicited high locomotor demands, particularly for high-speed running and sprinting. However, the magnitude of these responses varied according to the specific transition-game design and the combination of task constraints implemented. Asian-Clemente et al. [40] reported that TGs produced significantly greater distances across all speed zones than official match play for players in all positional groups. Asian-Clemente et al. [35] further showed that wider pitches (70 m) elicited greater high-speed running and sprint distances, whereas narrower pitches resulted in more accelerations. Rábano-Muñoz et al. [42] found that shorter defensive periods (30 s) produced higher high-speed running and sprint distances, while longer defensive periods (2 min) elicited more accelerations and higher ratings of perceived exertion (RPE). All included transition-game studies were classified as good methodological quality, strengthening confidence in the conclusion that these formats impose greater locomotor demands than conventional SSGs.

## 4. Discussion

This systematic review synthesized the evidence on the effects of six task-constraint categories on the physical, physiological and technical–tactical demands of soccer small-sided games (SSGs). The overall evidence demonstrates that each task constraint selectively modifies specific components of the training stimulus, with no individual constraint consistently reproducing the multidimensional demands of official match play. Rather, the physical, physiological and technical–tactical responses observed during SSGs emerge from the combined effects of interacting task constraints. Consequently, the design of SSGs should be based on the coordinated manipulation of pitch dimensions, player numbers, goalkeeper conditions, goal types, numerical configurations and transition-game formats according to the intended training outcome, rather than relying on the modification of a single constraint. It should also be recognized that the available evidence is not evenly distributed across outcome domains. Most included studies examined acute physical and physiological responses, whereas substantially fewer investigated technical–tactical outcomes. Consequently, the conclusions relating to technical–tactical adaptations should be interpreted more cautiously because they are supported by a comparatively limited evidence base.

### 4.1. Pitch Dimensions

Amongst the task constraints examined, pitch dimensions and the area per player (ApP) had the most consistent influence on the physical and physiological demands of SSGs. Larger playing areas increased external load by promoting higher total distance, high speed running and in the majority of studies, higher cardiovascular responses. However, the present review further points out that the effects of ApP cannot be considered in isolation from other task constraints. Riboli et al. [6] showed that the ApP required to replicate match demands varied considerably with the presence of a goalkeeper, pointing to the relationship between pitch dimensions and game format. Similarly, Castillo-Rodríguez et al. [36] determined an ApP of approximately 150–250 m^2^/per player was required to reproduce the physical and physiological demands of U19 match play; however, this threshold is context-specific and should not be interpreted as an optimal SSG design, as excessively large playing areas may reduce the player interactions that characterize SSGs.

The present findings also indicate that manipulating pitch dimensions alone cannot overcome the inherent limitations of SSGs in reproducing maximal sprinting and worst-case scenario (WCS) demands. Although increasing ApP increases high-speed running exposure, even large-ApP SSGs consistently fail to replicate the peak sprinting demands observed during official matches [37,43]. From a practical perspective, practitioners should combine moderate-sized SSGs with transition games or sprint-specific training, rather than expecting a single SSG format to simultaneously satisfy all physical and technical–tactical training objectives.

### 4.2. Player Number

The effects of player number on SSG demands appear to depend strongly on the spatial configuration and ApP. When ApP was controlled, smaller formats such as 4v4 elicited greater acceleration and deceleration demands and higher blood lactate concentrations, whereas larger formats such as 8v8 promoted greater very-high-speed running [15]. Similarly, across formats ranging from 4v4 to 10v10, smaller games imposed greater mechanical demands, while larger formats provided greater high-speed running exposure; however, no format fully reproduced match high-speed running demands [4]. Importantly, studies in youth players indicate that ApP may exert a stronger influence on locomotor demands than player number itself [16,17]; a finding further supported by the absence of substantial differences in internal load when ApP was maintained constant across 3v3, 5v5, and 8v8 formats [34]. Therefore, player number should not be considered an isolated constraint but interpreted in conjunction with ApP and other game-design characteristics. In addition, continuous rather than breakdown SSG bouts may enhance selected technical outcomes and reduce mood disturbance, although these effects reflect bout organization rather than player number alone [44].

### 4.3. Goalkeeper Presence and Role in SSG Design

Goalkeeper exclusion generally increases the physiological and locomotor demands imposed on outfield players during small-sided games, although the magnitude of this effect appears to depend on the spatial configuration of the game [19]. In particular, goalkeeper presence reduces outfield workload primarily on smaller pitches, with its influence diminishing as playing area increases [19]. This interaction is further supported by evidence showing that game format (e.g., possession-based versus goalkeeper-scoring conditions) may exert a greater influence on physical load than pitch size itself [29]. Collectively, these findings reinforce a central theme of this review: the effects of individual task constraints should not be considered in isolation, as goalkeeper conditions interact with spatial and rule-based constraints to shape the resulting training stimulus. Beyond these structural manipulations, goalkeeper verbal encouragement during small-sided games has also been associated with greater improvements in technical performance following a longitudinal training intervention [41], suggesting that goalkeeper behavior may represent an additional coaching-related constraint distinct from goalkeeper presence or absence.

### 4.4. Goal Type as a Regulator of SSG Demands

Goal type is an important task constraint for regulating the physical, physiological, and tactical demands of small-sided games, with its effects interacting with other elements of game design [7,14,18,24,28]. Mini-goal formats can reduce external load, including total distance, high-speed running, maximal speed, and high-intensity accelerations, while maintaining comparable heart-rate responses and neuromuscular fatigue [28]. However, these responses depend on game configuration. Small goals elicited higher heart-rate responses than regular goals during 4v4 but not 6v6 games [14], while pitch dimensions also modified the workload associated with different goal conditions [7]. Beyond physical demands, scoring targets influence collective tactical behavior, including team dispersion, spatial organization and space creation [18]. Therefore, mini-goals and alternative scoring formats may help coaches regulate locomotor exposure while maintaining technical–tactical involvement [24,28]. Nevertheless, goal type should be manipulated alongside player number, pitch dimensions and goalkeeper conditions rather than considered an isolated training constraint [7,14,24].

### 4.5. Numerical Imbalance and Floater Use: Practical Implications

Numerical imbalance and floater inclusion substantially modify the physical, physiological, and tactical demands of SSGs, although their effects depend on player role and game configuration [8,27,34]. Numerical inferiority generally increases physiological and locomotor demands, with outnumbered players performing more sprinting and high-intensity activity, particularly when greater playing areas are available [13,27,34]. Conversely, numerical superiority tends to reduce physical demands by providing players with greater opportunities to maintain possession and reposition at lower intensities [27]. Floater roles produce a distinctly lower workload than regular-player roles, with differences influenced by pitch size, goalkeeper inclusion and game format [8,38].

This reduced exposure supports the use of floaters for progressive workload management during return-to-play, including the staged floater-to-regular progression proposed by Lozano et al. [26]. Beyond physical demands, numerical superiority and floater inclusion modify exploratory behavior, offensive width, defensive compactness and spatial organization [20,21]. Therefore, numerical configurations should be selected according to both conditioning and tactical objectives rather than considered solely as a means of manipulating physical intensity. Most evidence in this category is derived from acute experimental studies examining immediate locomotor and tactical responses. Consequently, the long-term effectiveness of manipulating numerical imbalance remains uncertain.

### 4.6. Transition Games as a High-Intensity Training Format

Unlike the preceding categories, transition games should be regarded as a composite SSG format in which multiple task constraints including spatial configuration, numerical relationships, transition rules, and offensive–defensive organization are manipulated simultaneously. Consequently, the observed physical, physiological, and technical–tactical responses should be interpreted as the combined effects of these interacting constraints rather than the isolated influence of a single task constraint. Within this conceptual framework, the available evidence indicates that transition games consistently elicit greater locomotor demands than conventional SSG formats, particularly for high-speed running and sprinting, reflecting the combined influence of these interacting task constraints rather than any single design characteristic.

The TGs provide a distinct locomotor stimulus characterized by repeated high-speed actions within game-representative situations [39,40,42]. Unlike single-constraint manipulations, TGs combine spatial, numerical and transition-related conditions, and their demands therefore depend on the specific format applied. This is particularly relevant because conventional SSGs may provide insufficient high-speed running exposure relative to match play [4,5]. In contrast, TGs can substantially increase high-speed running and sprinting, although the magnitude of this response varies by playing position and game configuration [40]. Pitch width is an important moderator: wider TGs increase high-speed running, sprint distance and maximal speed, whereas narrower configurations promote more accelerations [35]. Similarly, transition structure and opponent positioning modify peak velocity, high-speed activity and perceived exertion [39]. Defensive-period duration also alters the stimulus, with shorter periods increasing high-speed running and sprinting, while longer periods increase acceleration demands and RPE [42]. Therefore, TGs provide a complementary format for targeting high-speed exposure, but their configuration should be systematically adjusted according to the intended locomotor stimulus and overall training load [5,35,39,42].

### 4.7. Practical Framework for Task Constraint Selection

The findings of this review indicate that task constraints should be selected and combined according to the primary objective of the training session because individual manipulations produce different, and often interacting, physical, physiological and technical–tactical responses [3,6,7]. No single SSG configuration appears capable of reproducing the multidimensional demands of competition. Consequently, practitioners should consider SSG design as an integrated process in which pitch dimensions, player number, goalkeeper conditions, scoring rules, numerical relationships and transition structure are coordinated to target specific outcomes.

When greater high-speed running exposure is required, increasing ApP generally provides more space for players to reach higher running velocities [6,31,36,43]. Studies have reported that ApP values approaching 150–250 m^2^ per player may reproduce selected match-related physical responses in youth players [36]. However, these values should be interpreted as experimental thresholds rather than recommended SSG prescriptions because they may substantially reduce player interaction density and shift the exercise toward a large-sided or match-simulation format. Moreover, even large-ApP formats may fail to reproduce peak match sprint demands [37,43]. Consequently, practitioners should avoid attempting to satisfy all physical objectives within a single SSG format. Moderate-sized SSGs should primarily be used to develop integrated physical, technical, and tactical qualities, whereas transition games and isolated sprint training should complement SSGs when the objective is to increase high-speed running, maximal sprint exposure, or worst-case scenario preparation [5,35,40].

Conversely, smaller formats and playing areas tend to emphasize accelerations, decelerations, and mechanical work rather than maximal-speed activity [4,15,37,43]. Goalkeeper conditions and numerical relationships provide additional means of regulating intensity and goalkeeper exclusion can increase outfield workload, particularly on smaller pitches [19], while numerical inferiority increases physiological and sprint-related demands [13,27,34]. Within transition games, wider pitches promote high-speed running and sprinting, whereas narrower configurations increase acceleration frequency; shorter defensive periods favor high-speed and sprint activity, while longer periods increase acceleration demands and perceived exertion [35,42].

Constraints can also facilitate workload management. Mini-goal formats can reduce locomotor load while maintaining comparable internal responses [28], whereas floaters typically experience lower physical demands than regular players [8,26], supporting their integration into progressive return-to-play strategies [26]. Importantly, these manipulations simultaneously influence tactical behavior: floater inclusion modifies offensive width and defensive compactness [21], while scoring targets alter team dispersion and spatial organization [18]. Accordingly, constraint selection should prioritize the principal session objective while recognizing physical, physiological and technical–tactical responses as interconnected outcomes. Finally, because the reviewed evidence is predominantly derived from male players, these practical recommendations should be generalized to female soccer with caution pending further sex-specific evidence.

### 4.8. Limitations

There are several limitations to be considered when interpreting the results of this review. First, there was a large heterogeneity between study designs, participant characteristics, small-sided game formats, task-constraint manipulations and outcome measures, which precluded quantitative synthesis and limited the ability to compare studies directly. Second, the current evidence base is dominated by investigations of the acute response, with only two studies examining longitudinal adaptations with randomized controlled designs [41,43]. This limits conclusions on the long-term effectiveness of various task-constraint manipulations. Third, technical–tactical outcomes were reported in a relatively small number of studies (12 of 40) and the lack of standardized methods of assessment limited the integration of these findings. Finally, because the available evidence is derived predominantly from male participants, the generalizability of these findings to female soccer remains uncertain. Accordingly, the practical recommendations should be interpreted with caution when applied to female players until robust sex-specific evidence is available. Future research should prioritize well-designed longitudinal randomized controlled trials, adopt standardized measures of technical–tactical performance and include more diverse populations across sex, age groups and competitive standards to strengthen the evidence base for task-constraint manipulation in soccer small-sided games.

## 5. Conclusions

This systematic review is the comprehensive synthesis of the impact of six major task-constraint categories on the physical, physiological and technical–tactical demands of soccer small-sided games. The results suggest that individual task constraints influence specific aspects of the training stimulus in a specific way and that no single constraint or SSG format can reproduce the multidimensional requirements of official match play. Thus, the responses to the training stimuli produced during SSGs are governed by the integration of different task constraints, which highlights the significance of considering the design of SSGs as a system of interrelated manipulations, rather than independent manipulations. From an applied perspective, findings support the intentional combining of pitch dimensions, area per player, player numbers, goalkeeper conditions, goal types, numerical configurations and transition-game formats according to the main goal of the training session. Transition-game formats appear to be an effective option for eliciting high locomotor demands, particularly for high-speed running and sprinting. However, because these formats combine multiple interacting task constraints, their effects should be interpreted within the context of their specific design characteristics rather than as evidence of a universally superior training stimulus. The findings provide an evidence-informed framework to assist practitioners design SSGs that better reflect the physical, physiological and technical–tactical demands of competition.

## Figures and Tables

**Table 1:** Methodological quality assessment of included studies using the Modified Downs and Black checklist.

Study	Constraint	R/7	E/3	B/7	C/6	Total/27	Grade
Casamichana et al. [12]	Player No.	7	2	6	4	19	Good
Torres-Ronda et al. [13]	Player No.	6	2	5	4	17	Good
González-Rodenas et al. [14]	Goal type	6	2	5	3	16	Fair
Rebelo et al. [15]	Player No.	7	2	6	4	19	Good
Castellano et al. [16]	Player No.	6	2	5	4	17	Good
Castellano et al. [17]	Player No.	6	2	5	4	17	Good
Castellano et al. [18]	Goal type	6	1	5	3	15	Fair
Hulka et al. [19]	Pitch/GK	6	2	5	4	17	Good
Torrents et al. [20]	Floater	5	1	4	3	13	Fair
Padilha et al. [21]	Floater	6	1	5	3	15	Fair
Lacome et al. [4]	Player No.	7	3	6	4	20	Good
Lacome et al. [22]	Floater	7	2	5	4	18	Good
Jara et al. [23]	GK role	6	2	5	3	16	Fair
Giménez & Gomez [24]	Goal type	6	2	5	3	16	Fair
Jara et al. [25]	GK role	6	2	5	3	16	Fair
Lozano et al. [26]	Floater	7	2	6	4	19	Good
Nunes et al. [27]	Floater	7	2	5	4	18	Good
Riboli et al. [6]	Pitch/GK	7	2	6	4	19	Good
Asian-Clemente et al. [5]	Transition	6	2	5	4	17	Good
Bujalance-Moreno et al. [28]	Goal type	7	2	6	5	20	Good
Modena et al. [7]	Pitch/Goal	6	2	5	4	17	Good
Santos et al. [29]	Pitch/GK	6	2	5	3	16	Fair
Santos et al. [30]	Pitch size	6	2	5	3	16	Fair
Mancini et al. [31]	Pitch size	6	2	5	3	16	Fair
Castillo et al. [32]	Pitch size	7	2	5	4	18	Good
Li et al. [33]	Player No.	7	2	6	4	19	Good
Guard et al. [34]	Floater	7	2	6	4	19	Good
Asian-Clemente et al. [35]	Transition	7	2	5	4	18	Good
Rábano-Muñoz et al. [8]	Floater	7	2	6	4	19	Good
Castillo-Rodríguez et al. [36]	Pitch size	7	2	6	5	20	Good
de Dios-Álvarez et al. [37]	Pitch size	7	2	6	5	20	Good
Asian-Clemente et al. [38]	Pitch size	7	2	5	4	18	Good
Asian-Clemente et al. [39]	Transition	6	2	5	4	17	Good
Asian-Clemente et al. [40]	Transition	7	2	5	4	18	Good
Wang et al. [9]	Pitch size	7	3	6	5	21	Good
Jumareng et al. [41]	GK role	7	3	6	5	21	Good
Rábano-Muñoz et al. [42]	Transition	7	2	5	4	18	Good
de Dios-Álvarez et al. [43]	Pitch size	7	2	6	5	20	Good
Farhani et al. [44]	Player No.	7	2	5	4	18	Good
Yilmaz et al. [45]	Pitch/No.	6	2	5	3	16	Fair

**Abbreviations**: B, Bias (internal validity); C, Confounding (internal validity); E, External validity; R, Reporting quality. Scores are presented as points obtained out of the maximum possible for each domain (see Section 2.2 for the modified Downs and Black checklist).

**Table 2:** Summary of studies examining pitch size and area per player.

Study	Population	SSG Format	Outcomes	Key Findings
Hulka et al. [19]	Junior males (*n* = 29)	5v5 ± GK; small/medium/large pitch	HR, distance, RPE	Larger pitch increased distance; GK reduces workload only on small pitch
Castillo et al. [32]	U16 males (*n* = 20)	5v5 + GK; 100 vs. 200 m^2^/player	Distance, accel/decel, sprints, impacts	LSG: more sprints, higher max velocity, greater body impacts
Riboli et al. [6]	Elite males (*n* = 25)	SSG ± GK; multiple area/player	TD, HI running, sprint, metabolic power	ApP to match demands: 187 m^2^ with GK vs. 115 m^2^ without; position-specific differences
Santos et al. [29]	U-23 sub-elite (*n* = 10)	4v4; possession vs. GK-scoring; 3 sizes	HR, player load, GPS	GK format type > pitch size effect on physical load; GK-scoring = stable internal load
Santos et al. [30]	U-12/U-15/U-23 (*n* = 24)	4v4; 3 pitch sizes (16 × 24 to 24 × 36 m)	Distance, metabolic power, TRIMP	Distance increases with pitch; metabolic power differs in U-12 only
Modena et al. [7]	Amateur males (*n* = 16)	3v3/4v4/5v5; 3 pitch sizes; ±GK	TD, HSR, HR, RPE	Larger pitch + no GK = highest demands; goal type × pitch interaction
Mancini et al. [31]	Amateur males (*n* = 18)	3v3/5v5/7v7; varied ApP	HR, distance, sprint zones	Sprint zone distances increase linearly with ApP; HR non-linear
de Dios-Álvarez et al. [37]	Professional males (*n* = 18)	4v4/6v6/8v8 + GK; varied ApP	GPS, worst-case scenario metrics	WCS 1 min demands exceeded all SSG formats; small SSGs overestimate accelerations, underestimate sprints
de Dios-Álvarez et al. [43]	Elite youth males (*n* = 16)	3 ApP conditions; multiple formats	GPS load metrics	Higher ApP: greater HSR/sprint; lower ApP: greater accel; no format replicated match WCS sprint demands
Castillo-Rodríguez et al. [36]	U19 males (*n* = 16)	Multiple SSG formats; 3 player numbers	HR, distance zones, physiological	150–250 m^2^/player threshold for match-like demands; below: sprint underload
Wang et al. [9]	Youth males (*n* = 24, RCT)	SSG elongated (2:1) vs. square pitch; 8 weeks	Sprint, endurance, agility	Elongated pitch: greater sprint improvements; square pitch: greater agility gains
Giménez & Gomez [24]	Professional males (*n* = 18)	3v3/5v5/7v7; SSG vs. circuit	HR, distance zones, RPE	3v3 highest HR and sprint; circuit produced more HSR time; SSG superior for physiological adaptations
Yilmaz et al. [45]	Amateur youth males (*n* = 22)	SSG with varied pre-SSG warm-up; 4 conditions	Technical performance, physical	10 min progressive warm-up optimal; improves passing accuracy and sprint without physiological penalty

**Abbreviations:** ApP, area per player; GK, goalkeeper; GPS, global positioning system; HI, high-intensity (running); HR, heart rate; HSR, high-speed running; LSG, large-sided game; *n*, number of participants; RCT, randomized controlled trial; RPE, rating of perceived exertion; SSG, small-sided game; TD, total distance; TRIMP, training impulse; WCS, worst-case scenario.

**Table 3:** Summary of studies examining the influence of player numbers on SSG demands.

Study	Population	SSG Format	Outcomes	Key Findings
Rebelo et al. [15]	Youth males (*n* = 24)	4v4 vs. 8v8 (same ApP)	Accel/decel, blood lactate, distance	4v4: more accel/decel (ES = 1.13–1.52), higher lactate; 8v8: more very-HSR
Lacome et al. [4]	Elite males (n = 21, PSG)	4v4 to 10v10; 249 SSG files	GPS: distance, HSR, sprint, accel	No format replicated match HSR; 4v4 overloads mechanical work; position × format interaction
Castellano et al. [16]	U9-U13 males (*n* = 36)	6v6 to 11v11; 3 goal types	Distance zones, HR, RPE	RPA > player number for locomotion; more players = higher max speed; goal type effect small
Castellano et al. [17]	U12-U13 males (*n* = 28)	7v7/9v9/11v11; 3 RPA	HR, distance, GPS metrics	RPA dominant predictor over player number in large-sided games; 7v7 at full-pitch RPA optimal
Li et al. [33]	Adult males (*n* = 16)	3v3/5v5/8v8; ApP fixed at 80 m^2^	HR, blood lactate, accel	No significant HR/lactate/accel differences when ApP held constant; ApP is primary intensity determinant
Casamichana et al. [12]	Amateur males (*n* = 18)	3v3/6v6/9v9; man-marking vs. free	HR, TD, player load, max speed	Man-marking: greater demands in 3v3/6v6; player number and marking both independently affect load
Torres-Ronda et al. [13]	Professional males (*n* = 14)	2v2/3v3/4v4/5v5 ± opponents	HR, time-motion, impacts	Reducing players: increase HR; increase impacts; 2v2: highest relative demands; max speed highest in larger formats
Farhani et al. [44]	Amateur males (*n* = 16)	3v3/5v5/7v7; continuous vs. fragmented	Technical performance, mood disturbance	Continuous 12 min > fragmented for passing, duels; lower mood disturbance; fewer players not always optimal

Abbreviations: accel, acceleration; ApP, area per player; decel, deceleration; ES, effect size; GPS, global positioning system; HR, heart rate; HSR, high-speed running; *n*, number of participants; PSG, Paris Saint-Germain (football club); RPA, relative pitch area; RPE, rating of perceived exertion; SSG, small-sided game; TD, total distance.

**Table 4:** Summary of studies examining goalkeeper presence and role.

Study	Population	SSG Format	Outcomes	Key Findings
Hulka et al. [19]	Junior males (*n* = 29)	5v5 ± GK; 3 pitch sizes	HR, distance, RPE	GK reduces outfield workload only on small pitches; no significant effect on larger pitches
Santos et al. [29]	U-23 sub-elite (*n* = 10)	4v4; possession vs. GK-scoring; 3 sizes	HR, player load, GPS	GK format type had greater effect than pitch size; GK-scoring = more stable physiological response
Riboli et al. [6]	Elite males (*n* = 25)	SSG ± GK; multiple ApP	TD, HSR, sprint, metabolic power	ApP to match TD: 187 m^2^ with GK vs. 115 m^2^ without; confirms GK × ApP interaction
Jara et al. [23]	Amateur males (*n* = 18, GKs)	3v3/4v4/5v5 + GK; 3 pitch sizes	GK technical actions, defensive distance	Larger pitch: more GK defensive actions; smaller formats: GK more technically active
Jumareng et al. [41]	Youth males (*n* = 32, 8-week RCT)	3v3 + GK with/without GK verbal encouragement	Technical performance, physical adaptations	GK encouragement increases passing, dribbling, shooting; amplified both physical and technical adaptations

Abbreviations: ApP, area per player; GK, goalkeeper; GPS, global positioning system; HR, heart rate; *n*, number of participants; RCT, randomized controlled trial; RPE, rating of perceived exertion; SSG, small-sided game; TD, total distance.

**Table 5:** Summary of studies examining the influence of goal type on SSG demands.

Study	Population	SSG Format	Outcomes	Key Findings
González-Rodenas et al. [14]	Youth males (*n* = 20)	4v4/6v6; regular vs. small goals vs. possession	HR, RPE, distance	Small goals increase HR vs. regular goals in 4v4 only; goal type × player number interaction confirmed
Bujalance-Moreno et al. [28]	Amateur males (*n* = 20)	6v6; possession vs. mini-goals	TD, HSR, max speed, accel, NMF	Mini-goals: lower external load; equivalent NMF; similar HR—selectively modulates volume
Castellano et al. [18]	Amateur males (*n* = 16)	5v5; small vs. regular goals; touch restriction	Dispersion, tactical shape metrics	Small-goal format: greater defensive openness, larger inter-team distance; shape metrics differ by goal type
Giménez & Gomez [24]	Professional males (*n* = 18)	3v3/5v5/7v7; SSG vs. circuit training	HR, distance zones, RPE	SSG with goal > possession for HSR; player number modulates goal-type effect on physiological response
Modena et al. [7]	Amateur males (*n* = 16)	3v3/4v4/5v5; 3 pitch sizes; regular vs. mini vs. no goal	TD, HR, RPE	Goal type × pitch size interaction; mini-goals preserve intensity while reducing distance at small pitches

Abbreviations: accel, acceleration; HR, heart rate; HSR, high-speed running; *n*, number of participants; NMF, neuromuscular fatigue; RPE, rating of perceived exertion; SSG, small-sided game; TD, total distance.

**Table 6:** Summary of studies examining numerical imbalance and floater use.

Study	Population	SSG Format	Outcomes	Key Findings
Padilha et al. [21]	Youth males (*n* = 22)	5v5 with/without floater; possession	Tactical shape metrics, heat map analysis	Floater: increase offensive width, increase defensive compactness; promoted numerical superiority exploitation
Torres-Ronda et al. [13]	Professional males (*n* = 14)	2v2/3v3/4v4/5v5 ± extra opponent	HR, time-motion, impacts	Reducing opponents: increase HR per player; adding opponents: increase sprinting and impacts for outnumbered team
Guard et al. [34]	Adult males (*n* = 16)	4v4/4v5/4v6; varied ApP	HR, GPS, RPE	4v6 (inferiority): increase mean HR (84.4 vs. 80.4% HRmax); inferiority amplified at higher ApP
Nunes et al. [27]	Youth males (*n* = 28)	4v2/4v4/4v6; possession format	HR, GPS, technical frequency	Dose–response: 4v6 increase sprints, tactical actions; 4v2 in crease walking; inferiority drives physical intensity
Torrents et al. [20]	Youth males (*n* = 20)	5v5/5v5 + 1/5v5 + 2; exploratory	Tactical behaviors, decision-making	Floater increases exploratory behaviors; numerical superiority promotes creative tactical solutions
Lozano et al. [26]	Youth males (*n* = 16)	SSG/LSG; floater vs. regular role	GPS, HR, match demands	Floaters: 40–60% less physical load than regular players; 4-stage RTP framework proposed
Rábano-Muñoz et al. [8]	Youth males (*n* = 18, 3 age groups)	5v5 + floater; age × role comparison	GPS, HR, metabolic power	Floater physical demands amplified in game-simulation formats; age × role interaction on sprint frequency
Asian-Clemente et al. [38]	Professional males (*n* = 25)	9v9 + 2 floaters; possession vs. position; 3 sizes	Distance, peak speed, PL, max accel/decel	Position games: lower DC/peak speed but higher max accel/decel; large pitch: greater demands

Abbreviations: accel, acceleration; DC, distance covered; decel, deceleration; GPS, global positioning system; HR, heart rate; LSG, large-sided game; *n*, number of participants; PL, player load; RPE, rating of perceived exertion; SSG, small-sided game.

**Table 7:** Summary of studies examining transition-game formats.

Study	Population	SSG Format	Outcomes	Key Findings
Asian-Clemente et al. [35]	Young professional males (*n* = 18)	3v2 TG; 3 pitch widths (30/50/70 m)	Distance at speed zones, peak speed, accel, RPE	TG70: greater HS distance, peak speed, sprint; TG30/50: higher accelerations; pitch width targets specific qualities
Asian-Clemente et al. [5]	Young professional males (*n* = 17)	5v5 + 5 floaters; SSG-CA vs. SSG-NC	TD, HS distance, VHSD, peak speed, accel vs. matches	SSG-CA: higher all metrics than SSG-NC; both exceeded match values for HSR/accel; matches: higher peak speed
Asian-Clemente et al. [39]	Young professional males (*n* = 18)	1v0/1v1-Front/1v1-Behind transition formats	Peak velocity, distance at speed zones, accel, RPE	1v1-Behind: highest peak velocity and DC ≥ 24 km/h; both 1v1 formats: higher RPE than 1v0
Rábano-Muñoz et al. [42]	Youth professional males (*n* = 14)	TG; 3 defensive period durations (30 s/1 min/2 min)	Distance at speed zones, accel, sprint, RPE	Short DP (30 s): increase HSR, sprint; longer DP (2 min): increase accel, RPE; DP duration shapes specific physical outputs
Asian-Clemente et al. [40]	Professional males (*n* = 20, by position)	Transition games vs. official matches	DC at multiple speed zones, peak speed, accel, PL	TGs exceeded match values across all positions; CBs/FBs/strikers showed greatest TG-vs-match discrepancy

Abbreviations: accel, acceleration; CA, change of playing area (SSG-CA); CB, center-back; DC, distance covered; DP, defensive period; FB, full-back; HS, high-speed (running); HSR, high-speed running; *n*, number of participants; NC, no change of playing area (SSG-NC); PL, player load; RPE, rating of perceived exertion; SSG, small-sided game; TD, total distance; TG, transition game; VHSD, very-high-speed distance.

## Data Availability

No new data were created or analyzed in this study. Data sharing is not applicable to this article.

## Supplementary Materials

The following supporting information can be downloaded at: https://www.mdpi.com/article/10.3390/jfmk11030354/s1, File S1: Full Review Protocol and Database Search Strategy; Table S1: Modifications to the original Downs and Black checklist and the rationale for each modification.
