# Supplementary material for: Effects of Task Constraint Manipulation on Physical, Physiological and Technical–Tactical Demands of Small-Sided Games in Soccer: A Systematic Review
Source: J Funct Morphol Kinesiol. 2026 Sep 5;11(3):354. doi: 10.3390/jfmk11030354 (PMC13608831; doi:10.3390/jfmk11030354)
Supplement: Supplementary file 1 [file jfmk-11-00354-s001.zip › jfmk-4460030-supplementary.pdf]

Supplementary File S1

Full Review Protocol and Database Search Strategy

Manuscript ID: jfmk-4460030 — "Effects of Task-Constraint Manipulation on Physical, Physiological and Technical–Tactical Demands of Small-Sided Games in Soccer: A Systematic Review"

This supplementary file provides the full review protocol, including the complete database search strategy, in response to Reviewer 2 (Comment 3) and to support the record registered in PROSPERO ("Availability of full protocol" field). It expands on the summary methods reported in Section 2 of the main manuscript; no methodological content differs between the two—this file simply provides the level of detail needed for replication.

1. Review Objective

To comprehensively synthesize evidence on the effects of task-constraint manipulation across six categories—(1) pitch size/area per player, (2) player number, (3) goalkeeper presence and role, (4) goal type, (5) numerical imbalance and floater players, and (6) transition game formats—on the physical, physiological and technical–tactical demands of small-sided games (SSGs) in association football.

2. Eligibility Criteria (PICOS)

Population: male football (soccer) players of any age or competitive level.

Intervention/Exposure: small-sided game (SSG) or transition game (TG) format involving manipulation of at least one of the six task-constraint categories listed above.

Comparator: alternative SSG/TG configuration, official match play, or no comparator (single-arm descriptive studies were eligible, provided outcome data were reported).

Outcomes: physical (e.g., total distance, high-speed running, sprint distance, accelerations/decelerations), physiological (e.g., heart rate, blood lactate, RPE) or technical–tactical outcomes (e.g., passes, team dispersion, tactical actions).

Study design: experimental or observational design with ≥10 participants, published in a peer-reviewed journal.

Exclusion criteria: sports other than football (e.g., futsal), duplicate datasets, conference abstracts/theses, review articles, meta-analyses, and female-only cohorts.

3. Information Sources

Four electronic databases were searched: PubMed/MEDLINE, SPORTDiscus, Web of Science Core Collection, and Scopus, from database inception to December 2024. The reference lists of all eligible studies and of relevant review articles were additionally hand-searched, and forward citation tracking ("cited-by" search) was performed in Scopus and Web of Science to identify further eligible records; this step identified two studies published after the database search cutoff (see Section 2.1 of the main manuscript for dates).

4. Search Strategy

The search strategy combined controlled vocabulary (where available in a given database) with free-text terms across three concept blocks, combined with Boolean operators: (1) small-sided game terms, (2) football/soccer terms, and (3) task-constraint / outcome terms. The core free-text terms used across all databases were:

Block A (game format): "small-sided game" OR "transition game*" OR "SSG"*

Block B (sport): "football" OR "soccer"

Block C (constraint/outcome): "pitch size" OR "goalkeeper" OR "mini-goal" OR "floater*" OR "numerical imbalance" OR "player number*" OR "physical demand*" OR "training load" OR "GPS" OR "heart rate" OR "tactical*"*

The exact, executed search string for each database—including field tags, controlled-vocabulary terms (e.g., MeSH for PubMed), and any date/language filters applied—is given below.

Database	Exact search string executed
PubMed/MEDLINE	(soccer[tiab] OR football[tiab]) AND ("small-sided game*"[tiab] OR "small sided game*"[tiab] OR SSG[tiab] OR "transition game*"[tiab]) AND ("pitch size"[tiab] OR "field size"[tiab] OR "playing area"[tiab] OR "area per player"[tiab] OR goalkeeper[tiab] OR "mini-goal*"[tiab] OR floater*[tiab] OR "numerical imbalance"[tiab] OR "player number*"[tiab] OR "physical demand*"[tiab] OR "training load"[tiab] OR GPS[tiab] OR "heart rate"[tiab] OR tactical*[tiab]); limited 2015-01-01 to 2024-12-31
SPORTDiscus	(soccer OR football) AND ("small-sided game*" OR "small sided game*" OR SSG OR "transition game*") AND ("pitch size" OR "field size" OR "playing area" OR "area per player" OR goalkeeper OR "mini-goal*" OR floater* OR "numerical imbalance" OR "player number*" OR "physical demand*" OR "training load" OR GPS OR "heart rate" OR tactical*)
Web of Science Core Collection	TS = ((soccer OR football) AND ("small-sided game*" OR "small sided game*" OR SSG OR "transition game*") AND ("pitch size" OR "field size" OR "playing area" OR "area per player" OR goalkeeper OR "mini-goal*" OR floater* OR "numerical imbalance" OR "player number*" OR "physical demand*" OR "training load" OR GPS OR "heart rate" OR tactical*)
Scopus	TITLE-ABS-KEY((soccer OR football) AND ("small-sided game*" OR "small sided game*" OR SSG OR "transition game*") AND ("pitch size" OR "field size" OR "playing area" OR "area per player" OR goalkeeper OR "mini-goal*" OR floater* OR "numerical imbalance" OR "player number*" OR "physical demand*" OR "training load" OR GPS OR "heart rate" OR tactical*)

Per-database record counts: PubMed/MEDLINE n = 361; SPORTDiscus n = 669; Web of Science Core Collection n = 986; Scopus n = 1,231. Combined total across the four databases: n = 3,247. All 3,247 records were exported to a single reference-management library (EndNote) and deduplicated across databases, identifying 634 duplicate records, leaving 2,613 unique records screened, consistent with Figure 1 in the main manuscript. Note: the search strings above were reconstructed and re-ran during manuscript revision, as the original search session/export records could not be located; the original search (database inception to December 2024) is believed to have used an equivalent strategy, but the exact historical query syntax could not be verified retrospectively.

5. Study Selection Process

Records identified across the four databases (n = 3,247) were exported to a single EndNote library and deduplicated across the pooled record set, identifying and removing 634 duplicate records (n = 2,613 unique records screened). Following title/abstract screening, full-text articles were assessed for eligibility against the criteria in Section 2 above. See Figure 1 (PRISMA 2020 flow diagram) in the main manuscript for the complete record of records identified, screened, excluded (with reasons), and included.

Records were managed and de-duplicated using EndNote; no dedicated systematic review screening platform (e.g., Rayyan, Covidence) was used. Both title/abstract screening and full-text eligibility assessment were performed independently and in duplicate by two reviewers (P.C., W.S.); disagreements at either stage were resolved through discussion between the two reviewers until consensus was reached, and no case required adjudication by a third reviewer.

6. Data Extraction

A standardized, piloted data-extraction form was used to record: first author and publication year; participant characteristics (age, sex, competitive level, sample size); SSG/TG format and the specific task constraint(s) manipulated; outcome measures and instrumentation (e.g., GPS/LPS sampling rate); and principal findings. Data were extracted by one reviewer (P.C.) and independently verified against the source articles by a second reviewer (W.S.), with discrepancies resolved through discussion.

7. Quality / Risk-of-Bias Assessment

Methodological quality was assessed using a modified Downs and Black checklist [Downs & Black, 1998], adapted for SSG research, across four domains: reporting quality (7 points), external validity (3 points), internal validity—bias (7 points), and

internal validity—confounding (6 points), for a maximum of 27 points. Studies were classified as good (≥17 points), fair (11–16 points), or poor (≤10 points).

8. Synthesis Methods

Given substantial heterogeneity in study designs, populations, SSG formats and outcome measures, a narrative synthesis (not meta-analysis) was performed, organized by the six task-constraint categories described in Section 1.

9. Registration

This protocol is registered on PROSPERO.

This protocol was registered on PROSPERO on 2 August 2026 (registration number CRD420261433575).

Supplementary Table S1.

Modifications to the original Downs and Black checklist and the rationale for each modification

**Original Downs & Black item**	**Modification**	**Rationale**
Randomization	Excluded	Not applicable because most included studies were non-randomized.
Blinding	Excluded	Blinding was not feasible in SSG intervention studies.
Compliance	Excluded	Most studies investigated acute responses rather than interventions requiring compliance monitoring.
Follow-up	Excluded	Long-term follow-up was not applicable to acute experimental designs.
Remaining items	Retained	Applicable across experimental and observational studies.
